# Supplementary material for: Impact of a Prescription Support Tool to Improve Adherence to the Guidelines for the Prescription of Oral Antithrombotics: The Combi-AT Randomized Controlled Trial Using Clinical Vignettes
Source: J Clin Med. 2019 Nov 8;8(11):1919. doi: 10.3390/jcm8111919 (PMC6912577; doi:10.3390/jcm8111919)
Supplement: Supplementary file 1 [file jcm-08-01919-s001.zip › Additional files Zerah J Clin Med-proof.docx]

**Additional file 2: Description of the 30 clinical vignettes (CVs).**

| **CV** | **Type** | **Description** |
| --- | --- | --- |
| **1** | Single anticoagulant therapy | Non-valvular atrial fibrillation (86 years old) |
| **2** | Single anticoagulant therapy | Non-valvular atrial fibrillation and asymptomatic lower extremity artery disease (76 years old) |
| **3** | Single anticoagulant therapy | Non-valvular atrial fibrillation and asymptomatic carotid stenosis (76 years old) |
| **4** | Dual therapy | Non-valvular atrial fibrillation and ST elevation myocardial infarction with percutaneous coronary intervention 6 months ago (60 years old) |
| **5** | Single anticoagulant therapy | Valvular heart disease (mechanical aortic valve) 20 years ago and stable ischemic heart disease in the last 10 years (75 years old) |
| **6** | Single anticoagulant therapy | Non-valvular atrial fibrillation and valvular mitral bioprosthesis one year ago (65 years old) |
| **7** | Single antiplatelet therapy | Ischemic stroke 9 months ago, pulmonary embolism with a triggering factor 6 months ago (49 years old) |
| **8** | Single antiplatelet therapy | Stable ischemic heart disease with percutaneous coronary intervention 2 years ago (81 years old) |
| **9** | Single anticoagulant therapy | Non-valvular atrial fibrillation and stable ischemic heart disease with percutaneous coronary intervention 18 months ago (72 years old) |
| **10** | Dual antiplatelet therapy | Non-ST elevation acute coronary syndrome medically managed 6 months ago (65 years old) |
| **11** | Dual therapy | Non-valvular atrial fibrillation and non-ST elevation acute coronary syndrome medically managed 6 months ago (85 years old) |
| **12** | Dual antiplatelet therapy | Non-ST elevation acute coronary syndrome with percutaneous coronary intervention 6 months ago (80 years old) |
| **13** | Dual antiplatelet therapy | ST elevation myocardial infarction with percutaneous coronary intervention 3 months ago (77 years old) |
| **14** | Dual antiplatelet therapy | ST elevation myocardial infarction with fibrinolytic therapy and percutaneous coronary intervention 3 months ago (75 years old) |
| **15** | Triple therapy | ST elevation myocardial infarction with left ventricular thrombus and percutaneous coronary intervention 2 months ago (55 years old) |
| **16** | Single anticoagulant therapy | Non-valvular atrial fibrillation and ST elevation myocardial infarction with percutaneous coronary intervention 1 year ago (76 years old) |
| **17** | Dual therapy | Non-valvular atrial fibrillation and non-ST elevation acute coronary syndrome with percutaneous coronary intervention 8 months ago (82 years old) |
| **18** | Dual antiplatelet therapy | Non-ST elevation acute coronary syndrome with coronary artery bypass graft 6 months ago (65 years old) |
| **19** | Dual antiplatelet therapy | ST elevation myocardial infarction with coronary artery bypass graft 3 months ago (60 years old) |
| **20** | Dual therapy | Non-valvular atrial fibrillation and ST elevation myocardial infarction with coronary artery bypass graft 3 months ago (45 years old) |
| **21** | No AT treatment | Aortic bioprosthesis 6 months ago (78 years old) |
| **22** | Single antiplatelet therapy | Carotid stenosis with stent 6 months ago (76 years old) |
| **23** | Single antiplatelet therapy | Lower extremity artery disease with percutaneous revascularization 6 months ago (65 years old) |
| **24** | Dual antiplatelet therapy | Carotid stenosis with stent 2 months ago and ST elevation myocardial infarction with percutaneous coronary intervention 8 months ago (77 years old) |
| **25** | Single anticoagulant therapy | Non-valvular atrial fibrillation and lower extremity artery disease with percutaneous revascularization 6 months ago (48 years old) |
| **26** | Single anticoagulant therapy | Lower extremity artery disease with percutaneous revascularization one year ago and mechanical heart valve 20 years ago (75 years old) |
| **27** | Single antiplatelet therapy | Transcatheter aortic valve replacement 6 months ago (86 years old) |
| **28** | Single anticoagulant therapy | Aortic mechanical heart valve and transient ischemic attack 6 months ago (50 years old) |
| **29** | Single anticoagulant therapy C | Discovery during medical consultation: deep venous thrombosis with triggering factor (36 years old) |
| **30** | Dual therapy | Aortic mechanical heart valve 8 years ago and ST elevation myocardial infarction with percutaneous coronary intervention 6 months ago (60 years old) |

**Additional file 3: Example of a clinical vignette.**

At your medical consultation, you meet Mr R, 86 years old (weight: 81 kg, body mass index: 24 kg/m^2^). Mr R is a widower, a smoker (10 cigarettes a day, 50 pack-years) and is autonomous in all daily activities. He has no personal medical history and he takes no drug. His last biological test did not find any abnormalities (serum creatinine value: 77 µM/L, creatinine clearance using the Cockcroft-Gault formula: 70 mL/min).

He comes to see you in consultation because he has had palpitations with exercise for more than 1 week. You perform electrocardiography (ECG) in your office and you diagnose non-valvular atrial fibrillation. The biological assessment shows no particularities (particularly blood ionography and thyroid-stimulating hormone). Cardiac ultrasonography revealed a dilated left atrium with no valve abnormality.

**1) How many antithrombotic treatments will you prescribe during this consultation?**

- 0
- 1
- 2
- 3

**2) If you answered 0 to question 1, go to question 5. If not, which molecule(s) of antithrombotic(s) will you prescribe during this consultation?**

- Warfarin
- Rivaroxaban
- Apixaban
- Aspirin
- Clopidogrel

**3) At which dosage will you prescribe this(these) molecule(s)? (For each molecule checked on the previous question, it will appear):**

- Warfarin:
  - INR (International Normalized Ratio): 2-3
  - INR (International Normalized Ratio): 2.5-3.5
- Rivaroxaban
  - 15 mg per day
  - 20 mg per day
- Apixaban
  - 2.5 mg twice a day
  - 5 mg twice a day
- Aspirin
  - 75-100 mg per day
  - 300 mg per day
- Clopidogrel
  - 75 mg per day
  - 300 mg per day

**4) How long does the antithrombotic treatment prescribed in the previous question need to be continued?**

- 1 month
- 6 months
- 12 months
- For life

**5) On a scale of 0 to 10, what is your degree of confidence in the adequacy of your prescription in relation to the guidelines?**

**For the experimental group, after completion of the 3 clinical vignettes:**

**Regarding the prescription support tool, please note the following items from 0 (strongly disagree) to 10 (strongly agree):**

- The prescription support-tool helped me answer to the clinical vignettes:../10
- The prescription support-tool has modified the answers to clinical vignettes that I would have made spontaneously:../10
- The prescription support-tool is clear:../10
- The prescription support-tool is operational:../10
- The prescription support-tool is useful for practice:../10
- I would be ready to use this prescription support-tool:../10
- I would recommend the use of this prescription support-tool:../10

**Notes on the tool: What are the points of the prescription support tool that could be improved: useless information, missing information, presentation, etc?:**

**Additional file 4: Graphical presentation of missing data pattern.**


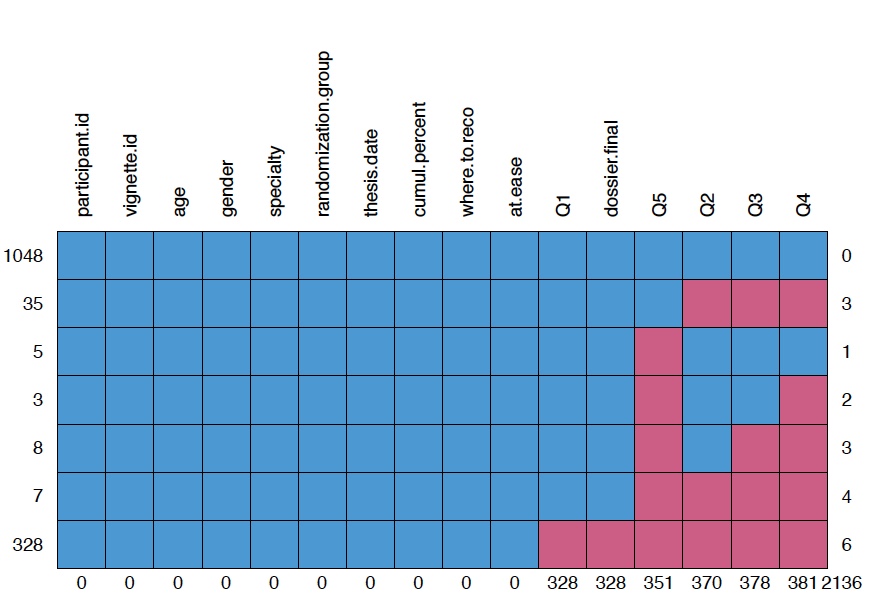


We considered that missing data were missing at random (MAR): “presence of missing values on a variable that is related to other observed variables but not related to its own unobserved values”.

We used data for all characteristics and all observed answers (questions 1 to 5) for the imputation modelling: package MICE (Multivariate Imputation by Chained Equations in R: <https://cran.r-project.org/web/packages/mice/mice.pdf)>.

Variables/abbreviations:

- Physicians’ characteristics: login (“participant.id”), age, sex, medical specialty, group of randomization (“randomization.group”), years of medical practice (“thesis.date”), average rate of their patients with prescription of oral AT combinations (“cumul.percent”), degree of self-confidence in prescribing oral AT combinations (“at.ease”). Finally, physicians were asked whether they knew the most recent guidelines on oral AT combinations and where to find them (“where.to.reco”).
- Four multiple-choice questions: question 1 (Q1): number of oral ATs; question 2 (Q2): type of oral ATs; question 3 (Q3): dosage of each oral AT prescribed; question 4 (Q4): duration of the prescription.
- The fifth and last question (Q5) evaluated the self-reported degree of confidence the physician had regarding the appropriateness of their prescription on a scale from 0 to 10.
- “Dossier.final” is a constructed variable representing the sum of Q1 to Q4 that is, the primary outcome (true if all questions were correct, false if one question or more were false). We reconstructed this variable after the multiple imputation.

**How to read the graphic?**

Y axes:

- 1048 clinical vignettes without missing data;
- 35 clinical vignettes with missing data for only questions 2, 3 and 4;
- 5 clinical vignettes with missing data for only question 5;
- 3 clinical vignettes with missing data for only questions 4 and 5;
- 8 clinical vignettes with missing data for only questions 3, 4 and 5;
- 7 clinical vignettes with missing data for only questions 2, 3, 4 and 5;
- 328 clinical vignettes with missing data for only questions 1, 2, 3, 4 and 5.

X axes:

- No missing data on physicians’ characteristics;
- 328 missing data on question 1;
- 351 missing data on question 5;
- 370 missing data on question 2;
- 378 missing data on question 3;
- 381 missing data on question 4;
- 2136 missing data for all data.

**Additional file 5: Comparison of baseline characteristics between physicians who completed at least one question for their three allocated clinical vignettes and those who did not complete any questions for their three allocated clinical vignettes. Values are numbers (percentages) unless stated otherwise.**

|  | **Did not complete any questions for their 3 allocated clinical vignettes**  **(N = 90)** | **Completed at least one question for their 3 allocated clinical vignettes**  **(N = 388)** | ***P*** |
| --- | --- | --- | --- |
| **Sex**  Male  Female | 44 (49)  46 (51) | 174 (45)  214 (55) | *P* = 0.56 |
| **Mean age (SD) (years)** | 44 (15) | 43 (13) | *P* = 0.38 |
| **Specialty**  GP  Cardiologist | 82 (91)  8 (9) | 379 (92)  29 (8) | *P* = 0.66 |
| **Randomization group**  Control arm  Experimental arm | 18 (20)  72 (80) | 222 (57)  166 (43) | *P* < 0.001 |
| **Years since graduation**  ≤ 1  2 to 5  6 to 10  11 to 20  ≥ 21 | 3 (3)  15 (17)  19 (21)  15 (17)  38 (42) | 11 (3)  96 (25)  90 (23)  64 (16)  127 (33) | *P* = 0.37 |
| **Percentage of patients taking oral AT combinations**  ≤5%  6 to 10%  11 to 20%  ≥ 21% | 53 (59)  22 (25)  12 (13)  3 (3) | 242 (62)  103 (27)  34 (9)  9 (2) | *P* = 0.49 |
| **How comfortable physicians feel with management of oral AT combinations**  Not comfortable at all  Rather uncomfortable  Rather comfortable  Very comfortable | 16 (18)  41 (46)  29 (32)  4 (5) | 73 (19)  206 (53)  102 (26)  7 (2) | *P* = 0.24 |
| **Guidelines about oral AT combinations: Do you know them and where to find them?**  No and I don’t know where to find them  No, but I know where to find them  Yes, and I know where to find them | 29 (32)  48 (53)  13 (15) | 176 (45)  148 (38)  64 (17) | *P* = 0.027 |

Abbreviations: AT: antithrombotic, GP: general practitioner.

**Additional file 6: Distribution of clinical vignettes between the two randomized groups of physicians. Values are numbers.**

**a) Intent-to-treat analysis with 478 physicians and 1434 clinical vignettes (CVs).**

| **CV** | **Type** | **Experimental group**  **(N = 714 CVs)** | **Control group**  **(N = 720 CVs)** |
| --- | --- | --- | --- |
| **1** | Single anticoagulant therapy | 24 | 24 |
| **2** | Single anticoagulant therapy | 24 | 23 |
| **3** | Single anticoagulant therapy | 25 | 25 |
| **4** | Dual therapy | 24 | 23 |
| **5** | Single anticoagulant therapy | 24 | 24 |
| **6** | Single anticoagulant therapy | 24 | 24 |
| **7** | Single antiplatelet therapy | 24 | 24 |
| **8** | Single antiplatelet therapy | 23 | 24 |
| **9** | Single anticoagulant therapy | 22 | 24 |
| **10** | Dual antiplatelet therapy | 23 | 24 |
| **11** | Dual therapy | 22 | 24 |
| **12** | Dual antiplatelet therapy | 24 | 24 |
| **13** | Dual antiplatelet therapy | 24 | 25 |
| **14** | Dual antiplatelet therapy | 24 | 24 |
| **15** | Triple therapy | 24 | 24 |
| **16** | Single anticoagulant therapy | 24 | 24 |
| **17** | Dual therapy | 24 | 24 |
| **18** | Dual antiplatelet therapy | 24 | 24 |
| **19** | Dual antiplatelet therapy | 24 | 23 |
| **20** | Dual therapy | 23 | 24 |
| **21** | No AT treatment | 24 | 24 |
| **22** | Single antiplatelet therapy | 24 | 24 |
| **23** | Single antiplatelet therapy | 24 | 23 |
| **24** | Dual antiplatelet therapy | 25 | 24 |
| **25** | Single anticoagulant therapy | 24 | 25 |
| **26** | Single anticoagulant therapy | 24 | 25 |
| **27** | Single antiplatelet therapy | 22 | 24 |
| **28** | Single anticoagulant therapy | 24 | 24 |
| **29** | Single anticoagulant therapy C | 24 | 23 |
| **30** | Dual therapy | 25 | 24 |

Abbreviations: CV: Clinical vignettes.

**b) Complete-case analysis with 388 physicians and 1106 clinical vignettes (CVs).**

| **CV** | **Type** | **Experimental group**  **(N = 469 CVs)** | **Control group**  **(N = 637 CVs)** |
| --- | --- | --- | --- |
| **1** | Single anticoagulant therapy | 15 | 23 |
| **2** | Single anticoagulant therapy | 12 | 20 |
| **3** | Single anticoagulant therapy | 18 | 23 |
| **4** | Dual therapy | 18 | 19 |
| **5** | Single anticoagulant therapy | 15 | 24 |
| **6** | Single anticoagulant therapy | 16 | 20 |
| **7** | Single antiplatelet therapy | 15 | 22 |
| **8** | Single antiplatelet therapy | 12 | 21 |
| **9** | Single anticoagulant therapy | 16 | 21 |
| **10** | Dual antiplatelet therapy | 16 | 22 |
| **11** | Dual therapy | 14 | 19 |
| **12** | Dual antiplatelet therapy | 15 | 22 |
| **13** | Dual antiplatelet therapy | 18 | 21 |
| **14** | Dual antiplatelet therapy | 19 | 20 |
| **15** | Triple therapy | 12 | 23 |
| **16** | Single anticoagulant therapy | 16 | 19 |
| **17** | Dual therapy | 12 | 22 |
| **18** | Dual antiplatelet therapy | 17 | 19 |
| **19** | Dual antiplatelet therapy | 19 | 21 |
| **20** | Dual therapy | 13 | 22 |
| **21** | No AT treatment | 15 | 22 |
| **22** | Single antiplatelet therapy | 20 | 21 |
| **23** | Single antiplatelet therapy | 17 | 19 |
| **24** | Dual antiplatelet therapy | 17 | 21 |
| **25** | Single anticoagulant therapy | 17 | 24 |
| **26** | Single anticoagulant therapy | 13 | 24 |
| **27** | Single antiplatelet therapy | 15 | 23 |
| **28** | Single anticoagulant therapy | 14 | 19 |
| **29** | Single anticoagulant therapy C | 17 | 20 |
| **30** | Dual therapy | 16 | 21 |

Abbreviations: CV: Clinical vignettes.

**Additional file 7: Sensitivity analyses.** Values are odds ratios (95% confidence intervals).

**a) Analyses with three different number of imputed datasets (M).**

| **Intent-to-treat analyses (N = 478 physicians and 1434 clinical vignettes)** | | | |
| --- | --- | --- | --- |
|  | **M = 5** | **M = 20**  **(results in the manuscript)** | **M = 50** |
| **Fully appropriate prescription (primary outcome)** | 3.57 [2.26 to 5.65]  *P* < 0.001* | 3.61 [2.60 to 5.02]  *P* < 0.001* | 3.98 [2.79 to 5.67]  *P* < 0.001* |
| **Fully appropriate prescription of oral ATs by type of prescriptions** | | | |
| **No AT treatment** | 3.54 [0.92 to 13.64]  *P* = 0.07 | 3.61 [0.96 to 13.51]  *P* = 0.06 | 3.45 [0.83 to 14.31]  *P* = 0.06 |
| **Single antiplatelet therapy** | 3.14 [1.63 to 6.07]  *P* < 0.001* | 2.89 [1.35 to 6.18]  *P* = 0.007* | 2.11 [1.03 to 4.33]  *P* = 0.004* |
| **Single anticoagulant therapy** | 2.35 [1.53 to 3.73]  *P* < 0.001* | 2.34 [1.47 to 3.72]  *P* < 0.001* | 2.36 [1.50 to 3.70]  *P* < 0.001* |
| **Dual antiplatelet therapy** | 2.75 [1.56 to 4.83]  *P* = 0.001* | 2.46 [1.42 to 4.26]  *P* = 0.001* | 2.42 [1.38 to 4.25]  *P* = 0.002* |
| **Dual therapy** | 14.80 [0.48 to 460.66]  *P* = 0.12 | 26.20 [0.25 to 2716.01]  P = 0.12 | 26.57 [0.05 to 13883.19]  *P* = 0.29 |
| **Triple therapy** |  |  |  |

**b) Complete-case analysis and modified intent-to-treat (ITT) analysis.**

|  | **Complete-case analysis**  **N = 388 physicians** | **Modified ITT analysis**  **N = 388 physicians** | |
| --- | --- | --- | --- |
| **Fully appropriate prescription (primary outcome)** | 3.98 [2.79 to 5.66]  *P* < 0.001* | 3.88 [3.04 to 4.95]  *P* < 0.001* | |
| **Fully appropriate prescription of oral ATs by type of prescriptions** | | |  |
| **No AT treatment** | 4.00 [0.99 to 16.15]  *P* = 0.05 | 3.54 [0.85 to 14.76]  *P* = 0.07 | |
| **Single antiplatelet therapy** | 4.07 [1.83 to 9.07]  *P* < 0.001* | 3.66 [1.71 to 7.84]  *P* < 0.001* | |
| **Single anticoagulant therapy** | 2.57 [1.61 to 4.11]  *P* < 0.001* | 2.61 [1.62 to 4.18]  *P* < 0.001* | |
| **Dual antiplatelet therapy** | 2.77 [1.52 to 5.05]  *P* < 0.001* | 2.81 [1.54 to 5.12]  *P* < 0.001* | |
| **Dual therapy** | 17.2 [0.20 to 1456.00]  *P* = 0.21 | 18.82 [0.28 to 1262.02]  *P* = 0.21 | |
| **Triple therapy** |  |  | |

Abbreviations: AT: antithrombotic. Intent-to-treat (ITT) = multiple imputations in a population involving all randomized physicians. Complete case = analysis without imputations involving physicians answering at least one question Modified ITT analysis: multiple imputations in a population involving physicians answering at least one question (20 datasets and seeds = 2019). Analysis involved a logistic mixed model for qualitative variables.

**Additional file 8: Complete-case analysis: compliance with guidelines of oral AT prescriptions by trial arm. Values are number (%) or odds ratio (OR) and 95% confidence interval (CI).**

|  | **Experimental arm**  **N = 166** | **Control arm**  **N = 222** | **OR [95%CI]** | ***P*** |
| --- | --- | --- | --- | --- |
| **All allocated clinical vignettes** | | | | |
| **Fully appropriate prescription** | 259 (55) | 186 (29) | 3.98 [2.79 to 5.66] | *P* < 0.001 |
| **Each component of the prescription**  Number of oral ATs  Type of oral ATs  Dosage of oral ATs  Duration of the prescription | 373 (80)  322 (72)  284 (88)  347 (79) | 382 (60)  325 (54)  236 (73)  375 (62) | 2.91 [2.12 to 4.00]  2.41 [1.78 to 3.26]  3.25 [2.27 to 7.94]  2.92 [2.18 to 4.85] | *P* < 0.001*  *P* < 0.001*  *P* < 0.001*  *P* < 0.001* |
| **Median (IQR) self-reported degree of confidence (/10)** | 7 [6 to 9] | 6 [4 to 8] | 4.29 [2.93 to 6.29] | *P* < 0.001* |
| **Clinical vignette with recommendation of no AT treatment** | | | | |
| **Fully appropriate prescription** | 9 (60) | 6 (27) | 4.00 [0.99 - 16.15] | *P* = 0.05 |
| **Number of oral ATs** | 9 (60) | 6 (27) | 4.00 [0.99 to 16.15] | *P* = 0.05 |
| **Median (IQR) self-reported degree of confidence (/10)** | 6 [5 to 8] | 6 [5 to 7] | 1.79 [0.43 to 7.48] | *P* = 0.41 |
| **Clinical vignettes with recommendation of single antiplatelet therapy** | | | | |
| **Fully appropriate prescription** | 55 (70) | 44 (42) | 4.07 [1.83 to 9.07] | *P* < 0.001* |
| **Each component of the prescription**  Number of oral ATs  Type of oral ATs  Dosage of oral ATs  Duration of the prescription | 72 (91)  63 (80)  59 (93)  63 (83) | 74 (70)  50 (48)  48 (96)  67 (64) | 5.16 [2.05 to 12.95]  4.97 [2.19 to 11.26]  1.23 [0.17 to 9.05]  2.92 [1.39 to 6.14] | *P* < 0.001*  *P* < 0.001*  *P* = 0.83  *P* = 0.004* |
| **Median (IQR) self-reported degree of confidence (/10)** | 7 [5 to 9] | 6 [4 to 7] | 3.52 [1.73-7.17] | *P* < 0.001* |
| **Clinical vignette with recommendation of single anticoagulant therapy** | | | | |
| **Fully appropriate prescription** | 84 (50) | 70 (30) | 2.57 [1.61 to 4.11] | *P* < 0.001* |
| **Each component of the prescription**  Number of oral ATs  Type of oral ATs  Dosage of oral ATs  Duration of the prescription | 124 (73)  110 (70)  94 (85)  135 (89) | 138 (58)  123 (57)  85 (69)  182 (84) | 2.15 [1.32 to 3.52]  2.17 [1.17 to 4.02]  3.82 [1.53 to 9.53]  1.63 [0.76 to 3.46] | *P* = 0.002*  *P* = 0.01*  *P* = 0.004*  *P* = 0.21 |
| **Median (IQR) self-reported degree of confidence (/10)** | 7 [6 to 8] | 6 [4 to 8] | 3.52 [2.13 to 5.83] | *P* < 0.001* |
| **Clinical vignette with recommendation of dual antiplatelet therapy** | | | | |
| **Fully appropriate prescription** | 77 (64) | 60 (41) | 2.77 [1.52 to 5.05] | *P* < 0.001* |
| **Each component of the prescription**  Number of oral ATs  Type of oral ATs  Dosage of oral ATs  Duration of the prescription | 103 (85)  93 (77)  90 (97)  90 (77) | 99 (68)  91 (65)  78 (86)  78 (55.7) | 3.10 [1.56 to 6.17]  1.92 [1.05 to 3.52]  5.18 [0.002 to 1.38 +04]  2.91 [1.38 to 6.11] | *P* = 0.001*  *P* = 0.03*  *P* = 0.68  *P* = 0.005* |
| **Median (IQR) self-reported degree of confidence (/10)** | 7 [7 to 9] | 6 [5 to 8] | 5.19 [2.92 to 9.23] | *P* < 0.001* |
| **Clinical vignette with recommendation of dual therapy** | | | | |
| **Fully appropriate prescription** | 34 (47) | 6 (6) | 17.2 [0.20 to 1456.0] | *P* = 0.2 |
| **Each component of the prescription**  Number of oral ATs  Type of oral ATs  Dosage of oral ATs  Duration of the prescription | 58 (80)  48 (67)  41 (85)  49 (69) | 53 (52)  50 (50)  24 (48)  42 (42) | 3.94 [1.94 to 8.02]  2.29 [1.14 to 4.60]  7.57 [2.51 to 22.87]  3.49 [1.60 to 7.63] | *P* < 0.001*  *P* = 0.02*  *P* < 0.001*  *P* = 0.002* |
| **Median (IQR) self-reported degree of confidence (/10)** | 7 [6 to 9] | 5 [4 to 7] | 5.56 [2.72 to 11.38] | *P* < 0.001* |
| **Clinical vignette with recommendation of triple therapy** | | | | |
| **Fully appropriate prescription** | 0 (0) | 0 () |  |  |
| **Each component of the prescription**  Number of oral ATs  Type of oral ATs  Dosage of oral ATs  Duration of the prescription | 7 (58)  7 (58)  0 (0)  10 (83) | 12 (52)  11 (48)  1 (9)  6 (26) | 1.28 [0.31 to 5.25]  1.53 [0.37 to 6.25]  14.17 [2.39 to 84.07] | *P* = 0.72  *P* = 0.56  *P* = 0.003* |
| **Median (IQR) self-reported degree of confidence (/10)** | 5 [2 to 7] | 5 [3 to 6] | 4.56 [0.71 to 29.20] | *P* = 0.11 |

Abbreviations: AT: antithrombotic, OR: odds ratio. CI: confidence interval; IQR, interquartile range. Analysis involved a logistic mixed model for qualitative variable and a linear mixed model for quantitative variables. Dosage of oral ATs: the percentage is given for physicians who chose the right type of oral ATs in the previous question.
